# Supplementary material for: Genetic structure of the small yellow croaker (Larimichthys polyactis) across the Yellow Sea and the East China Sea by microsatellite DNA variation: implications for the division of management units
Source: PeerJ. 2022 Aug 29;10:e13789. doi: 10.7717/peerj.13789 (PMC9435522; doi:10.7717/peerj.13789)
Supplement: Supplemental Information 1 [file peerj-10-13789-s001.zip › supplementary materials/Table S1.docx]

Table S1 Population identification of *L. polyactis* in the coastal waters of China based on different methods.

| Population division | Dividing evidence | References |
| --- | --- | --- |
| Three populations (the Yellow Sea and Bohai Sea, the South Yellow Sea, the East China Sea) | Migratory route | (Zhang et al., 1959) |
|  | Morphological differences | (Ikeda, 1964) |
|  | Spawning ground | (Hu, 1998) |
|  | Catch per unit effort | (Ying et al., 2011) |
|  | RAPD | (Meng et al., 2003) |
|  | AFLP | (Han et al., 2009, Lin et al., 2009) |
|  | Restriction-site Associated DNA | (Zhang et al., 2019) |
| Four populations (the Yellow Sea and Bohai Sea, the Yellow Sea, the South Yellow Sea, the East China Sea) | Geographical distribution | ([Lin](https://xueshu.baidu.com/s?wd=author%3A%28%E6%9E%97%E6%96%B0%E6%BF%AF%29%20%E4%B8%AD%E5%9B%BD%E6%B0%B4%E4%BA%A7%E7%A7%91%E5%AD%A6%E7%A0%94%E7%A9%B6%E9%99%A2%E4%B8%9C%E6%B5%B7%E6%B0%B4%E4%BA%A7%E7%A0%94%E7%A9%B6%E6%89%80&tn=SE_baiduxueshu_c1gjeupa&ie=utf-8&sc_f_para=sc_hilight%3Dperson), 1985) |
|  | Fishery resources survey | (Liu, 1990) |
|  | Habitat | (Jin, 2005) |
| Two populations (the Bohai Sea and the North Yellow Sea, the South Yellow Sea and the East China Sea | Ecological environment, biological characteristics | (Xu et al., 2009) |
| No significant differentiation among *L. polyactis* populations | Mitochondrial DNA | (Xiao et al., 2009, Kim et al., 2012) |
|  | Microsatellite DNA | (Kim et al., 2012, Li et al., 2013) |
|  | Restriction-site Associated DNA | (Zhang et al., 2015；Zhang et al., 2020b) |
