# Supplementary material for: Genetic structure of the small yellow croaker (Larimichthys polyactis) across the Yellow Sea and the East China Sea by microsatellite DNA variation: implications for the division of management units
Source: PeerJ. 2022 Aug 29;10:e13789. doi: 10.7717/peerj.13789 (PMC9435522; doi:10.7717/peerj.13789)
Supplement: Supplemental Information 1 [file peerj-10-13789-s001.zip › supplementary materials/Table S2 .docx]

Table S2 Frequencies of the null alleles of the 12 microsatellite loci developed for *L. polyactis* in seven localities

| Locus | Null allele frequency | | | | | | |
| --- | --- | --- | --- | --- | --- | --- | --- |
|  | YT | RS | QD | LYG | YC | ZS | WZ |
| Lpo103 | 0.001 | 0.006 | 0.003 | 0.005 | 0.001 | 0.011 | 0.009 |
| Lpo104 | 0.012 | 0.016 | 0.009 | 0.013 | 0.015 | 0.015 | 0.012 |
| Lpo105 | 0.037 | 0.036 | 0.029 | 0.030 | 0.026 | 0.034 | 0.023 |
| Lpo106 | 0.015 | 0.013 | 0.013 | 0.016 | 0.015 | 0.014 | 0.013 |
| Lpo109 | 0.015 | 0.016 | 0.014 | 0.013 | 0.013 | 0.016 | 0.011 |
| Lpo110 | 0.020 | 0.016 | 0.013 | 0.014 | 0.013 | 0.012 | 0.017 |
| Lpo111 | 0.029 | 0.027 | 0.037 | 0.028 | 0.021 | 0.025 | 0.027 |
| Lpo112 | 0.013 | 0.013 | 0.012 | 0.012 | 0.013 | 0.011 | 0.012 |
| Lpo113 | 0.014 | 0.013 | 0.010 | 0.011 | 0.010 | 0.011 | 0.012 |
| Lpo114 | 0.012 | 0.013 | 0.009 | 0.013 | 0.013 | 0.012 | 0.015 |
| Lpo115 | 0.032 | 0.034 | 0.030 | 0.033 | 0.014 | 0.031 | 0.013 |
| Lpo116 | 0.036 | 0.028 | 0.029 | 0.037 | 0.029 | 0.038 | 0.029 |
