# Supplementary material for: Genetic structure of the small yellow croaker (Larimichthys polyactis) across the Yellow Sea and the East China Sea by microsatellite DNA variation: implications for the division of management units
Source: PeerJ. 2022 Aug 29;10:e13789. doi: 10.7717/peerj.13789 (PMC9435522; doi:10.7717/peerj.13789)
Supplement: Supplemental Information 1 [file peerj-10-13789-s001.zip › supplementary materials/Table S3.docx]

Table S3 Genetic diversity parameters based on all loci (AL) and loci removing null alleles (RNAL) (*H*_o_=observed heterozygosity; *H*_e_=expected heterozygosity; *PIC*=polymorphic information content).

| ID | Locality | Sampling date | Sampling size | *H*_O_ | | *H*_E_ | | *PIC* | |
| --- | --- | --- | --- | --- | --- | --- | --- | --- | --- |
|  |  |  |  | AL | RNAL | AL | RNAL | AL | RNAL |
| YT | Yantai | 2019.12 | 24 | 0.976 | 0.972 | 0.920 | 0.912 | 0.889 | 0.882 |
| RS | Rushan | 2019.04 | 24 | 0.977 | 0.971 | 0.916 | 0.910 | 0.889 | 0.883 |
| QD | Qingdao | 2019.08 | 24 | 0.971 | 0.970 | 0.918 | 0.915 | 0.894 | 0.889 |
| LYG | Lianyungang | 2019.08 | 24 | 0.981 | 0.975 | 0.919 | 0.913 | 0.891 | 0.887 |
| YC | Yancheng | 2020.10 | 24 | 0.978 | 0.973 | 0.919 | 0.912 | 0.894 | 0.886 |
| ZS | Zhoushan | 2019.08 | 24 | 0.980 | 0.976 | 0.924 | 0.920 | 0.903 | 0.899 |
| WZ | Wenzhou | 2020.11 | 24 | 0.970 | 0.968 | 0.923 | 0.921 | 0.897 | 0.863 |
