# Supplementary material for: Genetic structure of the small yellow croaker (Larimichthys polyactis) across the Yellow Sea and the East China Sea by microsatellite DNA variation: implications for the division of management units
Source: PeerJ. 2022 Aug 29;10:e13789. doi: 10.7717/peerj.13789 (PMC9435522; doi:10.7717/peerj.13789)
Supplement: Supplemental Information 1 [file peerj-10-13789-s001.zip › supplementary materials/Table S4.docx]

Table S4 The sequence information (Liu et al., 2014) and Genetic diversity of 7 localities by 12 microsatellite loci.

| Locus | Repeat motif | Primer sequence (5′–3′) | Size | Ta (℃) | localities | | | | | | | |
| --- | --- | --- | --- | --- | --- | --- | --- | --- | --- | --- | --- | --- |
|  |  |  |  |  |  | YT | RS | QD | LYG | YC | ZS | WZ |
| Lpo103 | (GT)10 | F: GCACATTGGTTCCCAGTTTA | 181–214 | 58 | *A* | 17 | 19 | 17 | 19 | 21 | 15 | 17 |
|  |  | R: GTTCTCCTCATTCTCCCTCT |  |  | *A_R_* | 12.712 | 13.077 | 17.000 | 14.546 | 20.212 | 15.000 | 17.000 |
|  |  |  |  |  | *H*_o_ | 1 | 1 | 1 | 1 | 1 | 1 | 1 |
|  |  |  |  |  | *H*_e_ | 0.9291 | 0.922 | 0.9291 | 0.9521 | 0.9495 | 0.9566 | 0.9512 |
|  |  |  |  |  | *PIC* | 0.903 | 0.8955 | 0.9028 | 0.9282 | 0.9255 | 0.9331 | 0.9271 |
| Lpo104 | (CA)11 | F: AAACCAACCAACGGCTTCCA | 279–340 | 58 | *A* | 15 | 13 | 9 | 11 | 12 | 10 | 11 |
|  |  | R: AGACCAACCTCCGGCTCCTG |  |  | *A_R_* | 11.815 | 10.043 | 9 | 9.027 | 10.232 | 10.000 | 11.000 |
|  |  |  |  |  | *H*_o_ | 1 | 1 | 1 | 1 | 1 | 1 | 1 |
|  |  |  |  |  | *H*_e_ | 0.9331 | 0.9432 | 0.9366 | 0.9562 | 0.9325 | 0.9233 | 0.9123 |
|  |  |  |  |  | *PIC* | 0.8978 | 0.8461 | 0.8587 | 0.8483 | 0.8995 | 0.8617 | 0.8625 |
| Lpo105 | (CA)19 | F: AGACAGACAAGGAGGAGTGA | 140–191 | 54 | *A* | 13 | 14 | 12 | 11 | 13 | 15 | 11 |
|  |  | R: CAAAGATAGATGACGAACCG |  |  | *A_R_* | 12.813 | 13.803 | 12.000 | 10.281 | 12.253 | 15.000 | 11.000 |
|  |  |  |  |  | *H*_o_ | 0.9333 | 0.9157 | 0.9032 | 0.9483 | 0.9321 | 0.9465 | 0.9112 |
|  |  |  |  |  | *H*_e_ | 0.8952 | 0.8865 | 0.9023 | 0.9237 | 0.9123 | 0.9163 | 0.9214 |
|  |  |  |  |  | *PIC* | 0.8521 | 0.8432 | 0.8998 | 0.8988 | 0.9012 | 0.9102 | 0.8932 |
| Lpo106 | (GT)19 | F: ACAAAGTAAACCACCGAGAC | 100–156 | 56 | *A* | 12 | 13 | 13 | 16 | 15 | 14 | 13 |
|  |  | R: CTGGAGCAAATGCAAATA |  |  | *A_R_* | 11.698 | 12.643 | 13.000 | 15.619 | 14.251 | 14.000 | 13.000 |
|  |  |  |  |  | *H*_o_ | 0.9465 | 0.9032 | 0.9231 | 0.9421 | 0.9231 | 0.9325 | 0.9023 |
|  |  |  |  |  | *H*_e_ | 0.9246 | 0.8777 | 0.8901 | 0.8812 | 0.8920 | 0.8927 | 0.8965 |
|  |  |  |  |  | *PIC* | 0.8838 | 0.8991 | 0.9236 | 0.8767 | 0.8895 | 0.9136 | 0.9120 |
| Lpo109 | (GT)23 | F: AAGCAGAACCATTGGAGTAT | 102–160 | 52 | *A* | 15 | 16 | 14 | 13 | 13 | 16 | 11 |
|  |  | R: CTGTCGATTCAGAAGAAAGA |  |  | *A_R_* | 14.606 | 15.856 | 14.000 | 12.756 | 12.654 | 16.000 | 11.000 |
|  |  |  |  |  | *H*_o_ | 0.9517 | 0.9435 | 0.9532 | 0.9222 | 0.9210 | 0.96667 | 0.9425 |
|  |  |  |  |  | *H*_e_ | 0.9205 | 0.9239 | 0.9398 | 0.9021 | 0.9202 | 0.9456 | 0.9078 |
|  |  |  |  |  | *PIC* | 0.9238 | 0.9062 | 0.9256 | 0.8852 | 0.8926 | 0.9268 | 0.8783 |
| Lpo110 | (GA)10 | F: CTTCAACATTTCCTCCATTT | 217-263 | 52 | *A* | 20 | 16 | 13 | 14 | 13 | 12 | 17 |
|  |  | R: GTGTTCAGGACTGCGTATTT |  |  | *A_R_* | 14.696 | 13.237 | 13.000 | 11.338 | 11.322 | 12.000 | 17.000 |
|  |  |  |  |  | *H*_o_ | 1 | 1 | 1 | 1 | 1 | 1 | 1 |
|  |  |  |  |  | *H*_e_ | 0.953 | 0.945 | 0.9406 | 0.9167 | 0.8927 | 0.9424 | 0.9450 |
|  |  |  |  |  | *PIC* | 0.9292 | 0.9204 | 0.9155 | 0.8891 | 0.8625 | 0.9176 | 0.9204 |
| Lpo111 | (GT)10 | F: GAGTCAGCAGTCAAATAGC | 179-206 | 52 | *A* | 15 | 20 | 17 | 18 | 21 | 15 | 17 |
|  |  | R: TGAAGCAACGATGAATAAG |  |  | *A_R_* | 11.271 | 13.823 | 17.000 | 13.805 | 17.256 | 15.000 | 17.000 |
|  |  |  |  |  | *H*_o_ | 1 | 1 | 1 | 1 | 1 | 1 | 1 |
|  |  |  |  |  | *H*_e_ | 0.9105 | 0.9371 | 0.9193 | 0.9459 | 0.9495 | 0.9530 | 0.9530 |
|  |  |  |  |  | *PIC* | 0.8819 | 0.912 | 0.8919 | 0.9214 | 0.9255 | 0.9292 | 0.9290 |
| Lpo112 | (GT)17 | F: TTTATTTGGGCACGAGATGT | 106-163 | 58 | *A* | 13 | 13 | 12 | 12 | 13 | 11 | 12 |
|  |  | R: GAACCCAGGAGTGCAGAATC |  |  | *A_R_* | 10.543 | 10.806 | 12.000 | 10.326 | 11.325 | 11.000 | 12.000 |
|  |  |  |  |  | *H*_o_ | 1 | 1 | 1 | 1 | 1 | 1 | 1 |
|  |  |  |  |  | *H*_e_ | 0.9246 | 0.8777 | 0.8901 | 0.8812 | 0.9113 | 0.8927 | 0.9096 |
|  |  |  |  |  | *PIC* | 0.8691 | 0.8726 | 0.8637 | 0.8827 | 0.8827 | 0.8898 | 0.8808 |
| Lpo113 | (CA)8…(CA)7 | F: CAAGGTGCCTACTTTGTGA | 194-238 | 58 | *A* | 14 | 13 | 10 | 11 | 10 | 11 | 12 |
|  |  | R: ACTGCTGAATAAGTTATGTCC |  |  | *A_R_* | 11.063 | 10.165 | 10.000 | 9.395 | 9.654 | 11.000 | 12.000 |
|  |  |  |  |  | *H*_o_ | 0.9583 | 1 | 0.875 | 0.9583 | 1 | 0.9167 | 0.8752 |
|  |  |  |  |  | *H*_e_ | 0.9167 | 0.9007 | 0.9025 | 0.8954 | 0.8723 | 0.8998 | 0.9007 |
|  |  |  |  |  | *PIC* | 0.8887 | 0.8706 | 0.8728 | 0.8642 | 0.8394 | 0.8705 | 0.8704 |
| Lpo114 | (CA)18 | F: ACAGGATCACAGGCATTTCC | 129-177 | 52 | *A* | 12 | 13 | 9 | 13 | 13 | 12 | 15 |
|  |  | R: ACGAGCATTTCATGACGAGT |  |  | *A_R_* | 10.024 | 10.631 | 9.000 | 10.968 | 11.252 | 12.000 | 15.000 |
|  |  |  |  |  | *H*_o_ | 1 | 1 | 1 | 1 | 1 | 1 | 1 |
|  |  |  |  |  | *H*_e_ | 0.9087 | 0.9113 | 0.9122 | 0.9113 | 0.9122 | 0.9078 | 0.9246 |
|  |  |  |  |  | *PIC* | 0.8795 | 0.8827 | 0.8834 | 0.8831 | 0.8839 | 0.8789 | 0.8979 |
| Lpo115 | (GT)12 | F: AAACTCACTGTGATGGTTGT | 192-264 | 54 | *A* | 13 | 14 | 10 | 13 | 14 | 11 | 13 |
|  |  | R: AAATAGCCTGACACTTCTTG |  |  | *A_R_* | 10.125 | 11.627 | 10.000 | 11.022 | 12.252 | 11.000 | 13.000 |
|  |  |  |  |  | *H*_o_ | 0.9167 | 0.9583 | 1 | 1 | 0.9583 | 1 | 1 |
|  |  |  |  |  | *H*_e_ | 0.8945 | 0.9264 | 0.9096 | 0.9176 | 0.9300 | 0.9096 | 0.9131 |
|  |  |  |  |  | *PIC* | 0.8638 | 0.8997 | 0.8805 | 0.89 | 0.9036 | 0.8805 | 0.8848 |
| Lpo116 | (CA)10 | F: AGGCACAAATGATTGAACTT | 300–364 | 52 | *A* | 17 | 18 | 19 | 17 | 19 | 15 | 19 |
|  |  | R: AGAATGAAGCCAAGAAGTGA |  |  | *A_R_* | 12.037 | 13.37 | 19.000 | 13.497 | 17.232 | 15.000 | 19.000 |
|  |  |  |  |  | *H*_o_ | 1 | 1 | 1 | 1 | 1 | 1 | 1 |
|  |  |  |  |  | *H*_e_ | 0.9246 | 0.9424 | 0.9379 | 0.9468 | 0.9495 | 0.9521 | 0.9362 |
|  |  |  |  |  | *PIC* | 0.8977 | 0.9175 | 0.9128 | 0.9223 | 0.9253 | 0.9284 | 0.9107 |
