# Supplementary material for: Genetic structure of the small yellow croaker (Larimichthys polyactis) across the Yellow Sea and the East China Sea by microsatellite DNA variation: implications for the division of management units
Source: PeerJ. 2022 Aug 29;10:e13789. doi: 10.7717/peerj.13789 (PMC9435522; doi:10.7717/peerj.13789)
Supplement: Supplemental Information 1 [file peerj-10-13789-s001.zip › supplementary materials/Table S5.docx]

Table S5 Pairwise *R*_ST_ among *L. polyactis* localities (*Significant p<0.005 after Bonferroni correction for multiple comparisons)

| Population | YT | RS | QD | LYG | YC | ZS | WZ |
| --- | --- | --- | --- | --- | --- | --- | --- |
| YT |  |  |  |  |  |  |  |
| RS | 0.0075 |  |  |  |  |  |  |
| QD | 0.0069 | 0.0013 |  |  |  |  |  |
| LYG | -0.0054 | 0.0029 | 0.0047 |  |  |  |  |
| YC | 0.0063 | -0.0078 | 0.0035 | -0.0095 |  |  |  |
| ZS | 0.0004* | 0.0079 | 0.0145 | -0.0075* | 0.0109* |  |  |
| WZ | 0.0016 | -0.0083 | 0.0123 | -0.0080 | 0.0054 | 0.0056 |  |
