# Supplementary figures and images for: Genetic structure of the small yellow croaker (Larimichthys polyactis) across the Yellow Sea and the East China Sea by microsatellite DNA variation: implications for the division of management units
Source: PeerJ. 2022 Aug 29;10:e13789. doi: 10.7717/peerj.13789 (PMC9435522; doi:10.7717/peerj.13789)

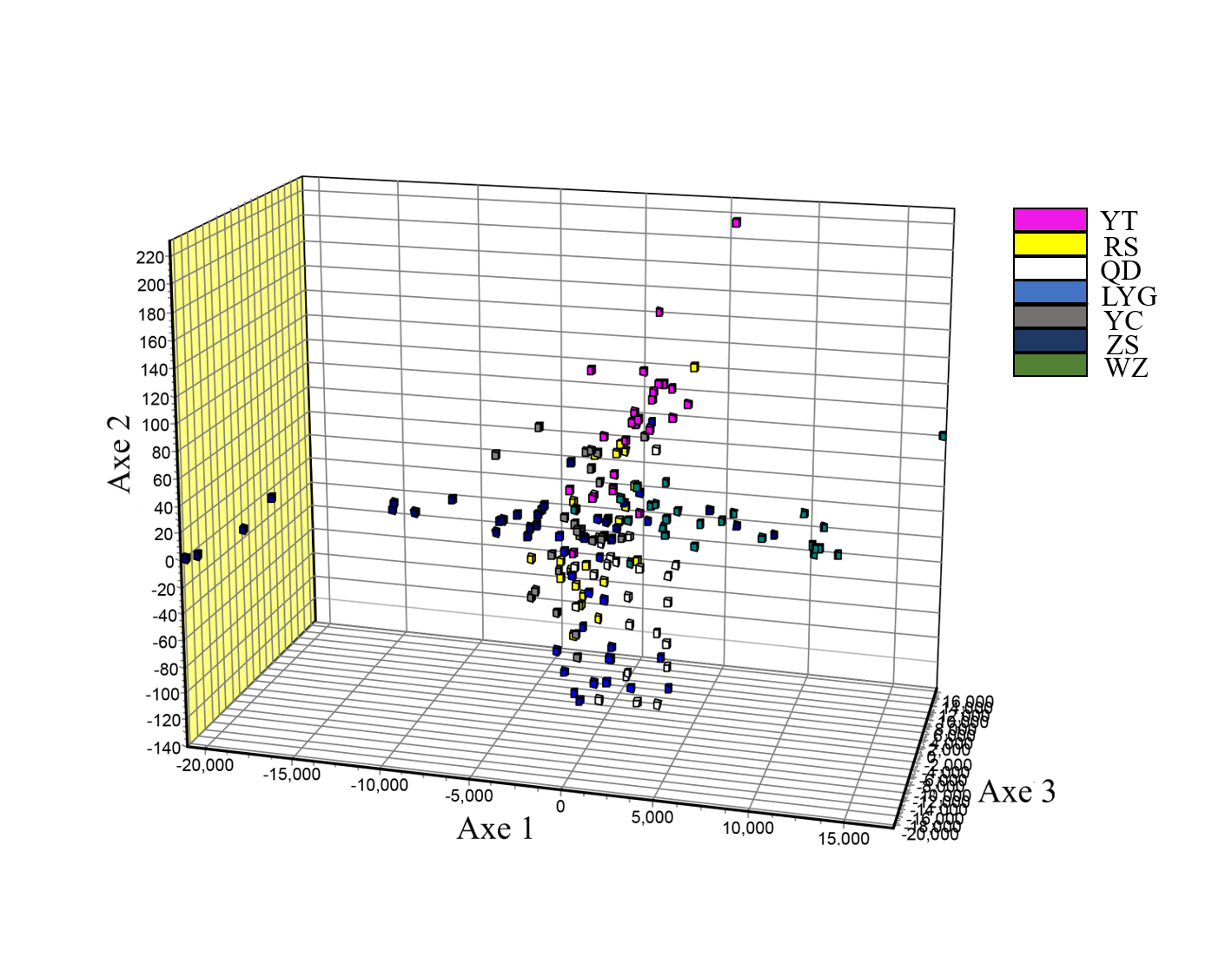

Supplement: Supplemental Information 1 [file peerj-10-13789-s001.zip › supplementary materials/Fig S1.tif]
