# Supplementary material for: Genetic structure of the small yellow croaker (Larimichthys polyactis) across the Yellow Sea and the East China Sea by microsatellite DNA variation: implications for the division of management units
Source: PeerJ. 2022 Aug 29;10:e13789. doi: 10.7717/peerj.13789 (PMC9435522; doi:10.7717/peerj.13789)
Supplement: Supplemental Information 2 [file peerj-10-13789-s002.zip › raw data 1/README.docx]

The raw microsatellite data is provided in this part. In our study, forward microsatellite primers were 5′ -labelled with a fluorescent dye (HEX, FAM or TAMRA). This data can be opened by the software of Genemarker v.1.91. The fluorescent dye of microsatellite primers and populations are as follow:

| Data | 5′ -labelled | populations |
| --- | --- | --- |
| Lpo103, Lpo104, Lpo105 P1 | Lpo103: HEX,  Lpo104: FAM  Lpo105: TAMRA | (1)-(24): YT  (25)-(48): RS  (49)-(72): QD  (73)-(96): LYG |
| Lpo103, Lpo104, Lpo105 P2 | Lpo103: HEX,  Lpo104: FAM  Lpo105: TAMRA | (1)-(24): YC  (25)-(48): ZS  (49)-(72): WZ |
| Lpo106, Lpo109, Lpo110 P1 | Lpo106: HEX,  Lpo109: FAM  Lpo110: TAMRA | (1)-(24): YT  (25)-(48): RS  (49)-(72): QD  (73)-(96): LYG |
| Lpo106, Lpo109, Lpo110 P2 | Lpo106: HEX,  Lpo109: FAM  Lpo110: TAMRA | (1)-(24): YC  (25)-(48): ZS  (49)-(72): WZ |
| Lpo111, Lpo112, Lpo113 P1 | Lpo111: HEX,  Lpo112: FAM  Lpo113: TAMRA | (1)-(24): YT  (25)-(48): RS  (49)-(72): QD  (73)-(96): LYG |
| Lpo111, Lpo112, Lpo113 P2 | Lpo111: HEX,  Lpo112: FAM  Lpo113: TAMRA | (1)-(24): YC  (25)-(48): ZS  (49)-(72): WZ |
| Lpo114, Lpo115, Lpo116 P1 | Lpo114: HEX,  Lpo115: FAM  Lpo116: TAMRA | (1)-(24): YT  (25)-(48): RS  (49)-(72): QD  (73)-(96): LYG |
| Lpo114, Lpo115, Lpo116 P2 | Lpo114: HEX,  Lpo115: FAM  Lpo116: TAMRA | (1)-(24): YC  (25)-(48): ZS  (49)-(72): WZ |
